# Contribution to the Preclinical Safety Assessment of *Lannea velutina* and *Sorindeia juglandifolia* Leaves

**DOI:** 10.3390/plants12010130

**Published:** 2022-12-27

**Authors:** Quintino Malú, Katelene Lima, Maryam Malmir, Rui Pinto, Isabel Moreira da Silva, Luís Catarino, Maria Paula Duarte, Rita Serrano, João Rocha, Beatriz Silva Lima, Olga Silva

**Affiliations:** 1 Research Institute for Medicines (iMed.ULisboa), Faculty of Pharmacy, Universidade de Lisboa, 1649-003 Lisbon, Portugal; 2 Dr Joaquim Chaves Laboratório de Análises Clínicas, 2790-224 Carnaxide, Portugal; 3 Centre for Ecology, Evolution and Environmental Changes, (cE3c) & CHANGE-Global Change and Sustainability Institute, Faculty of Sciences, Universidade de Lisboa, 1749-016 Lisboa, Portugal; 4 MEtRICs/Chemical Department, Nova School of Science and Technology, Universidade Nova de Lisboa, 2829-516 Caparica, Portugal

**Keywords:** Ames test, genotoxicity, *Lannea velutina*, repeated-dose oral toxicity, *Sorindeia juglandifolia*

## Abstract

Dried leaves of *Lannea velutina* A. Rich. and *Sorindeia juglandifolia* (A. Rich.) Planch. ex Oliv. (family Anacardiaceae) are used in African traditional medicine. Although these medicinal plants have widespread use in the treatment of inflammatory diseases, there is no scientific data concerning their preclinical or clinical safety. This work aimed to investigate the phytochemical properties of the leaves of both species using HPLC-UV/DAD, as well as the in vivo oral repeated-dose toxicity of 70% hydroethanolic leaf extract of *S. juglandifolia* and the in vitro genotoxicity of 70% hydroethanolic leaf extracts of *L. velutina* and *S. juglandifolia*. Clinical signs of toxicity, body weight variations, and changes in food consumption, mortality, and blood biochemical parameters were monitored. Genotoxicity was assessed using the bacterial reverse mutation assay (Ames test) with and without metabolic activation, according to OECD guidelines. The obtained results showed the presence of gallic acid and anacardic acid as the main marker constituents in both species. No significant changes in general body weight or food intake were observed; small significant changes with no critical relevance were observed in the blood biochemistry of animals treated with *S. juglandifolia* hydroethanolic extract (50, 400, and 1000 mg/kg body weight) compared to those in the control group. No genotoxicity was observed in the bacterial reverse mutation assay with *S. juglandifolia* and *L. velutina* extracts (up to 5 mg/plate). The safety data obtained in vivo and lack of genotoxic potential in vitro points to the safe medicinal use of *S. juglandifolia* and *L. velutina* extracts.

## 1. Introduction

The Anacardiaceae R.Br. family comprises approximately 80 genera and 800 species, distributed mainly in tropical or subtropical regions. Due to their economic importance as a source of food, valuable timber, and medicinal compounds, members of this family are cultivated worldwide [1]. This family, composed mainly of trees and shrubs, is well known for species that produce a poisonous sap that induces contact dermatitis (*Comocladia*, *Metopium*, *Semecarpus*, *Toxicodendron* spp.), lacquer plants (*Toxicodendron* and *Gluta* spp.), and particularly for its cultivated edible fruits and seeds (mangos, pistachios, and cashews), as well by other numerous species that produce edible fruits and seeds, such as *Lannea* spp. and *Sorindeia* spp. [2].

*Lannea velutina* and *Sorindeia juglandifolia*, commonly known as “mantede” in the archipelago of Bijagós (Guinea-Bissau), are two Anacardiaceae species native to tropical Africa [3,4,5,6] that are widely used in traditional medicine in Guinea-Bissau and countries such as Senegal, Guinea-Conakry, Gambia, Sierra Leone, Côte d’Ivoire, Cameroon, Sudan, the Democratic Republic of Congo, Mozambique, and Angola [4,5,6].

Different plant parts of *L. velutina* (leaf, stem, bark, and root) have been used in traditional medicine to treat various ailments, including skin diseases, fever, gastrointestinal diseases [6], diarrhea, rachitis [6,7], external pain, back pain, and nerve pain [8,9], dermatitis, dysentery, chronic gastric ulcer, boils, inflammations, and hemorrhoids [7].

Phytochemical studies of the *Lannea* genus have identified phenolic compounds, flavonoids, alkylphenols, dihydrocylcohexenones, dihydrocylcohexenols, and alkylated hydroquinones [10].

Several authors have shown many in vitro biological activities of *L. velutina* leaf, bark, and root, including antibacterial [6,11,12], fungicidal, larvicidal [11], molluscicidal, antioxidant [12], and anti-inflammatory activities [5]. Extracts of other *Lannea* species also reportedly have in vitro antioxidant, anti-inflammatory, analgesic, acetylcholinesterase inhibitory activity, antimalarial, anti-HIV, antibacterial, antifungal, and antiviral activities [10].

Concerning *Sorindeia juglandifolia*, the fruit is reportedly used in traditional medicine to treat children’s liver disease and mouth sores [13]. Some compounds, such as 2,3,6-trihydroxy benzoic acid and 2,3,6-trihydroxy methyl benzoate, were identified and isolated from the fruits of *S. juglandifolia* [4]. Vitexin, flavones, catechins, stigmasterol, methyl gallate-related compounds, and tachioside were identified and isolated from the leaves of *S. juglandifolia* [14]. Some authors also reported the presence of phenolic compounds (flavonoids, gallotannins, tannins), saponins, anthraquinones, coumarins, terpenoids, alkaloids, sterols, and fatty acids in aqueous extracts of *S. juglandifolia* and *S. grandifolia* leaves [15,16]. The few pharmacological studies found in the literature reported mainly the in vitro and in vivo antiplasmodial and antimalarial activities of the whole plant, its fractions, and isolated and purified compounds 2,3,6-trihydroxy benzoic acid and 2,3,6-trihydroxy methyl benzoate [4], as well as the antimycobacterial activity of these same compounds isolated from the fruit of *S. juglandifolia* [13,17].

Although *L. velutina* and *S. juglandifolia* are widely used in traditional African medicine, little information is found in the literature concerning their chemical composition, pharmacological activities, or preclinical safety.

According to WHO (World Health Organization), medicinal plants and traditional medicines with established quality, efficacy, and safety profiles can significantly contribute to healthcare access for millions of people. Herbal medicines, traditional treatments, and traditional healers/practitioners are the primary source of healthcare and sometimes the only source of care for many people. Alongside their affordability, these traditional medicine systems are accessible, culturally accepted, and trusted by many people [18,19].

These traditional systems are usually accepted as reliable and safe sources of healthcare because of their long history of usage in several cultures [20]. However, the absence of evidence of side effects and toxicity does not necessarily mean safety [21]. The long history of usage of these plants in traditional medicine is not a guarantee of safety, as traditional healers/practitioners, most of the time, are not able to detect or monitor effects such as rare adverse effects or adverse effects resulting from long-term usage (genotoxicity, cancer, etc.) [22]. Therefore, because of the general but mistaken conviction that natural products are harmless [21,23], toxicological evaluations of medicinal plants have been overlooked for many years [22]. Because of the lack of specific investigation related to medicinal plants’ toxicity, only severe and acute adverse effects are usually identified [21].

Guidelines describing the assessment of toxicity and genotoxicity of pharmaceuticals have been established by OECD (Organization for Economic Cooperation and Development), ICH (International Conference on Harmonization), and EMA (European Medicines Agency) committees. The preliminary assessment of the toxicity and genotoxicity of pharmaceuticals involves a battery of toxicity studies and genotoxicity tests [24,25]. However, according to the Committee on Herbal Medicinal Products (HMPC) “many herbal substances and preparation that are included in well-established products and or traditional herbal medicinal products (HMPs) have an acceptable safety profile, supported or at least partially substituted by their documented history of long-term medicinal and/or food use” [26,27]. For this reason, special attention should be given to adverse effects that are difficult or not possible to detect through clinical assessment, such as genotoxicity, carcinogenicity, and toxicity to reproduction [26,27].

Therefore, this research aimed to characterize the short-term repeated oral toxicity of *S. juglandifolia* hydroethanolic extracts and the genotoxicity of *L. velutina* and *S. juglandifolia* hydroethanolic extracts.

To the best of our knowledge, this is the first study to assess the genotoxicity of *L. velutina* and *S. juglandifolia* hydroethanolic extracts.

## 2. Results and Discussion

### 2.1. HPLC-UV/DAD Analysis

**Figure 1:**
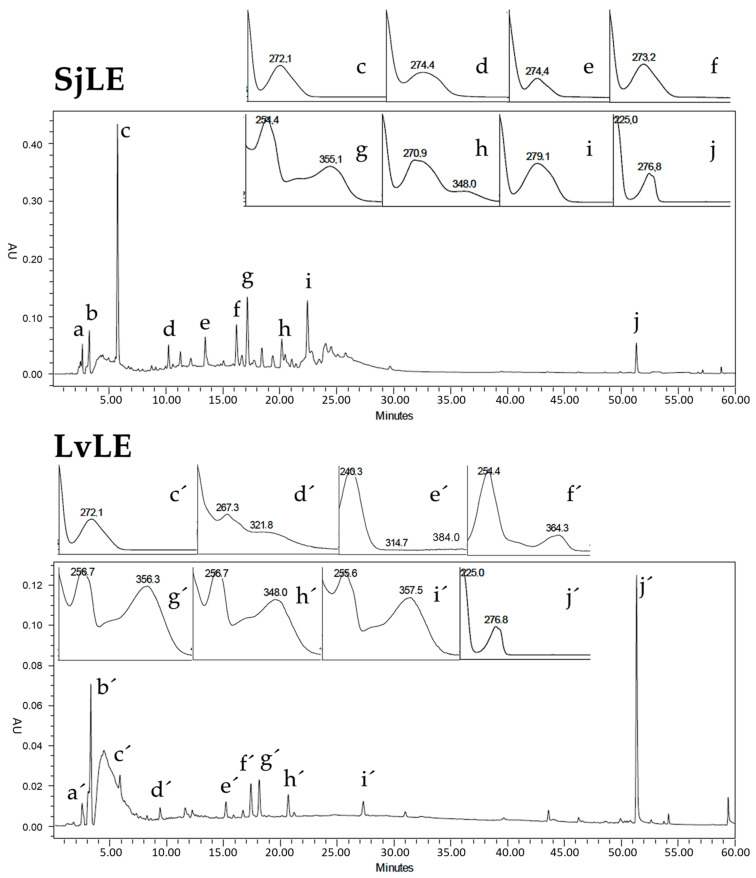
Comparative HPLC-UV/DAD chromatographic profiles of secondary metabolites of *S. juglandifolia* and *L. velutina* extracts.

A comparative analysis of the retention times and ultraviolet photodiode array (HLPC-UV/DAD) spectra of standards with the obtained peaks of the respective chromatograms showed the presence of gallic acid (c,c′), and anacardic acid (j,j′) in both *S. juglandifolia* 70% hydroethanolic extract (SjLE) and *L. velutina* 70% hydroethanolic extract (LvLE) (Figure 1). Identification of these compounds was also confirmed using co-chromatography data. Gallic acid (c) was detected as the major marker compound of *S. juglandifolia*, and anacardic acid (j′) was detected as the major marker compound of *L. velutina* extracts. A more detailed analysis of the obtained *S. juglandifolia* chromatogram showed the presence of phenolic compounds, particularly phenolic acids, and hydrolyzable tannins. In *L. velutina*, phenolic acids and flavonoids (quercetin derivatives) were evident.

### 2.2. Repeated Dose Toxicity (28 Days)

**Figure 2:**
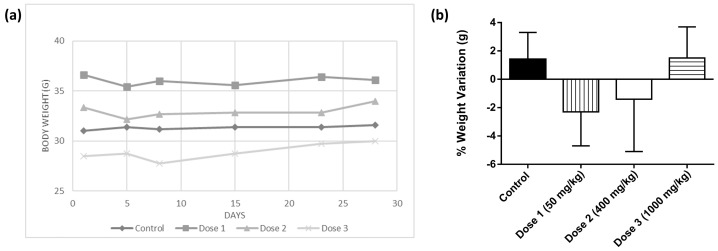
Body weight variation (**a**) and percentage of body weight variation (**b**) in mice administered by gavage with different doses of *S. juglandifolia* leaf extract.

SjLE was administered daily in single doses of 50, 400, and 1000 mg/kg bw for 28 days to CD–1 mice. The statistical analysis of the data revealed no significant differences (*p* < 0.005) in the % weight variation in the three groups administered SjLE compared to the control group (Figure 2, Table S1 of Supplementary materials). In fact, these results were very similar to previous results obtained by Alide et al. [15] in which treatment of animals with a decoction of *S. juglandifolia* twigs and leaves significantly prevented a decrease in body weight.

**Figure 3:**
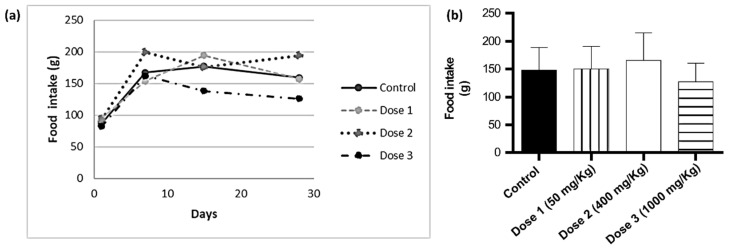
Cumulative food intake (**a**) and mean variation of food intake (**b**) of mice administered by gavage with different doses of *S. juglandifolia* leaf extract.

As for the statistical analysis of the cumulative food intake, there were no significant differences (*p* < 0.005) between the SjLE–treated and control groups (Figure 3, Table S1 of supplementary materials). Regarding the evaluation of clinical and behavioral signs, there were four deaths, all resulting from misadministration (confirmed with necropsy after each death) and not related to the toxicity of the extract. During the experiment, SjLE–treated animals did not show clinical changes compared to those in the control group. The necropsy and macroscopic examinations of the organs revealed no relevant changes except for diarrhea in the animals treated with the highest dose of SjLE (1000 mg/kg) in the last days of treatment (Table S2 of supplementary materials).

**Figure 4:**
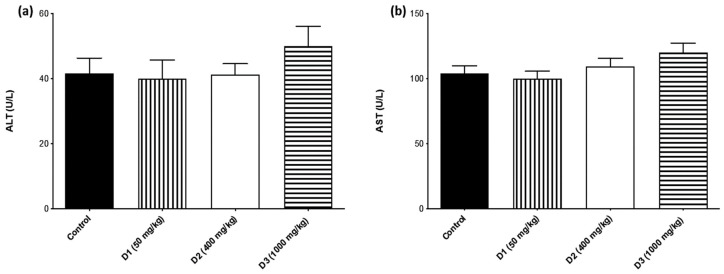
Liver injury biomarkers. Evaluation of the expression of (**a**) ALT and (**b**) AST in mice administered by gavage with different doses of *S. juglandifolia* leaf extract and comparison with the control group.

**Figure 5:**
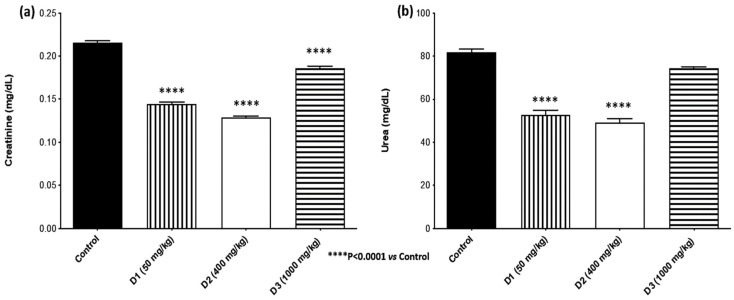
Renal function biomarkers. Evaluation of the expression of (**a**) creatinine and (**b**) urea in mice administered by gavage with different doses of *S. juglandifolia* leaf extract and comparison with the control group.

Serum biomarkers of crucial importance in evaluating the safety of SjLE (50, 400, and 1000 mg/kg bw) were analyzed. The results showed that liver biomarkers AST and ALT of the SjLE–treated groups did not present significant changes compared to the control group (Figure 4), indicating that the three tested doses did not cause hepatotoxicity. Regarding the rena creatinine and urea, significant changes were observed between the SjLE–treated and control groups (Figure 5). The serum levels of creatinine and urea in all treated groups presented lower levels than in the control group. However, these lower results were not interpreted as an indication of toxicity since the control group (which received only water) also showed lower values compared to historical control values (0.28–0.37 mg/dL) [28,29]. The observation of decreased values of creatinine led us to discard the presence of toxicity since only increased creatinine levels are considered indicative of kidney injury [30]. Furthermore, a dose-effect relationship was not observed regarding this decrease, which also excluded a direct toxic effect. Additionally, it is not unusual for extracts containing phenolic acids to induce a diuretic effect [31], which can be responsible for minor variations in increased kidney function without any relation to toxicity. This aspect was corroborated by Grácio et al., who reported a similar variation in creatinine serum values with the induction of hyperglycemia in the same species/strain of animals without damaging effects.

**Figure 6:**
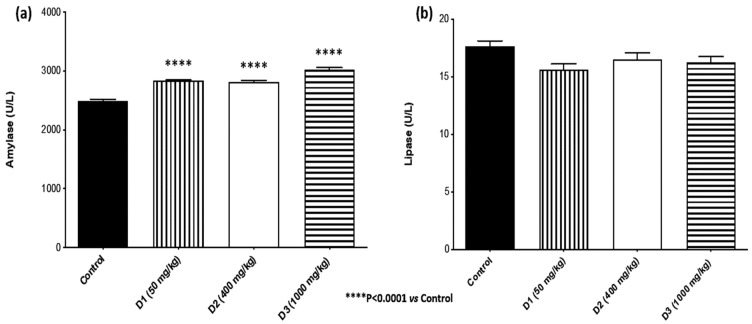
Pancreatic injury biomarkers. Evaluation of the expression of (**a**) amylase and (**b**) lipase in mice administered by gavage with different doses of *S. juglandifolia* leaf extract and comparison with the control group.

For the studied pancreatic biomarkers (Figure 6), the results revealed no significant differences between the SjLE–treated and control groups for lipase. Still, it was possible to observe a dose-dependent increase in enzyme levels compared to the control group for amylase. The observation of increased amylase values was not considered a sign of pancreatic toxicity since the margin of toxicity for this parameter is considerably higher than that for other parameters. In this type of experiment, the variations were approximately 10–15%, which waswithin the normal variation for animal experimentation [28]. The obtained results showed small variations in amylase values compared to the control group, along with normal values of serum lipase, evidencing the absence of pancreatic toxicity. Our findings agreed with the study by Grácio et al. in which amylase values of control animals of the same species/strain (~3000 U/L) were compared to double the amylase values of animals with hyperglycemia (~6000 U/L), a condition that is not pathological. For this reason, the small variations in the observed amylase values were not characterized as potential toxicity since these values could be attributed to the physiological variability of animals and not to a toxic effect. Observations of the pancreas in the necropsy examination did not reveal any toxic alterations.

**Figure 7:**
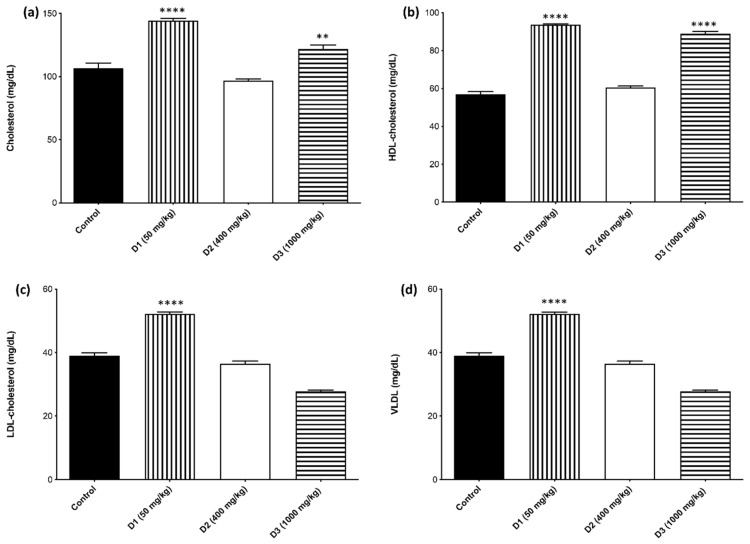

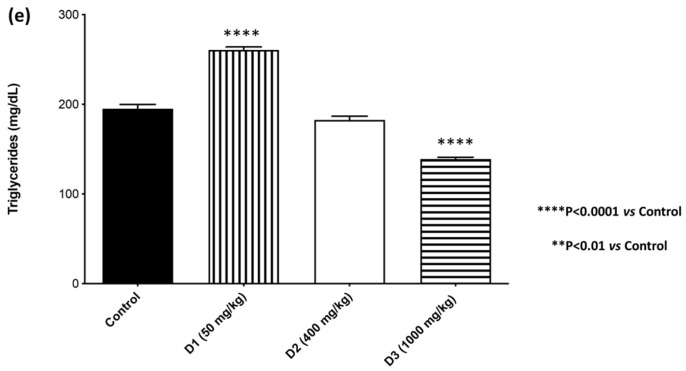
Lipid profile biomarkers. Evaluation of the expression of (**a**) cholesterol, (**b**) HDL, (**c**) LDL, (**d**) VLDL, and (**e**) triglycerides in mice administered by gavage with different doses of *S. juglandifolia* leaf extract and comparison with the control group.

The evaluation of the lipidic profile biomarkers indicated that for total cholesterol and HDL (Figure 7a,b), there were significant differences between the SjLE D1 and SjLE D3–treated groups compared to the control group. In contrast, the SjLE D2-treated group presented similar values to the control group. The increased HDL levels may have influenced the increase in TC levels, pointing to a potential cardiovascular beneficial effect of SjLE since, according to ref. [32], HDL helps extract cholesterol from cells.

Compared to the control group, a dose-dependent decrease in the levels of LDL, VLDL, and triglycerides (Figure 7c–e) was observed in all SjLE–treated groups. For the SjLE lower dose (D1), the levels of these biomarkers were increased; for the D2 group, the levels were like the control group; and for the higher dose (D3), the levels were lower than the levels of the control group.

These differences may be related to the high levels of HDL observed for the D1 and D3 groups, corroborating to some extent the potential hypocholesterolemia effects of the extract. In fact, a study by Alide et al. also reached similar conclusions, reporting that a leaf extract of *S. juglandifolia* was able to increase the HDL–cholesterol levels of animals treated with different doses and significantly reduced serum cholesterol, LDL–cholesterol, and triglyceride levels.

The results for the biochemical parameters were corroborated by the results obtained by Alide et al. in which *S. juglandifolia* extract also protected animals from a significant increase in transaminase activity, hypercholesterolemia, and hypercreatininemia in the kidney.

Apart from some significant changes that fell within the range of expected significance without toxicity implications, there was no evidence of toxicity in any of the biochemical parameters analyzed nor in any of the experimental SjLE doses tested.

The obtained results were also reinforced by a previous study in which the SjLE marker compound, gallic acid, showed a NOAEL (No Observed Adverse Effect Level) of 5000 mg/kg when given orally to mice [33].

### 2.3. Bacterial Reverse Mutation Assay (Ames Test)

The bacterial reverse mutation test (Ames test) is commonly employed as an initial screening for genotoxic/mutagenic potential [34]. The importance of risk assessment using simple toxicological tests, such as the Ames test, is currently a key standard for testing medicinal products established by OECD, ICH, and EMEA committees [35,36]. Additionally, according to the HMPC guidelines for herbal medicinal products/substances, evaluating genotoxicity initially using a bacterial reverse mutation test (Ames test) with different bacterial strains and metabolic activation is a standard requirement for submission for marketing authorization and application for simple registration [26]. This test uses strains of *Salmonella enterica* serovar Typhimurium, which are histidine auxotrophs, to detect point mutations (substitution, addition, or deletion of one or a few DNA base pairs) [36,37,38,39].

A substance or compound is considered genotoxic/mutagenic in the Ames test when it produces a reproducible dose-related increase in the number of revertant colonies for at least one of the tester strains. Moreover, the number of revertant colonies must be more than twofold the number of colonies produced on the solvent control plates, and cytotoxicity can be checked by investigation of the background layer of bacteria [27,37].

The Ames test was performed using five histidine auxotroph strains of *S.* Typhimurium (TA98, TA100, TA102, TA1535, and TA1537). According to the results, there was neither a dose-related increase in the number of revertant colonies nor a duplication of the number of revertant colonies at any concentration tested (250, 625, 1250, 2500, 3750, and 5000 µg/plate) for the extracts of *L. velutina* and *S. juglandigfolia* with and without metabolic system (Table 1 and Table 2, respectively) compared to the negative control. Evidence of cytotoxicity was not found, as there was no decrease in the background lawn of the plates or in the number of spontaneous revertants at any of the concentrations tested.

Therefore, under the conditions of this study, the results indicated that a mutagenic effect was not observed for any of the leaf extracts of *L. velutina* and *S. juglandifolia* tested up to the concentration of 5 mg/plate in any of the five strains tested, with and without metabolic activation.

Studies evaluating the genotoxic potential of medicinal plants are needed to identify plant species that may pose mutagenic and carcinogenic risks. In recent years, the number of studies using this type of assay to evaluate plant extracts’ genotoxic/mutagenic potential has increased [40]. Studies have reported that several plants used in traditional medicine are potentially genotoxic [40,41] and some of the species identified as genotoxic belong to the Anacardiaceae family (e.g., *Anacardium ocidentale*, *Taipirira guianensis*) [42], including one species from the genus *Lannea* [43]. According to these authors, *Lannea edulis* presented marginal genotoxicity in the strain TA97, but *Lannea discolor* did not [43].

To date, there is no information regarding the genotoxicity of the genus *Sorindeia*. The obtained results are the first to describe the genotoxic potential of *L. velutina* and *S. juglandifolia* leaves. This information is of the utmost importance since it reports the non-genotoxicity of these two medicinal plants with and without metabolic activation up to the maximum concentration recommended by OECD guidelines. Previous studies with gallic acid, a marker compound identified in both tested extracts, reported no mutagenic effects up to the concentration of 5000 µg/plate using the Ames test [44]. Additionally, gallic acid has been described as genoprotective and hepatoprotective [45].

## 3. Materials and Methods

### 3.1. Chemicals, Reference Items, and Metabolic Activation System

Ammonium sodium phosphate dibasic tetrahydrate, anhydrous dipotassium hydrogen phosphate, and sodium chloride were purchased from Fluka (Seelze, Germany). Bacto™ agar was purchased from Becton Dickinson & Co (Sparks, MD, USA). Citric acid monohydrate, disodium hydrogen phosphate dihydrate, and sodium dihydrogen phosphate monohydrate were purchased from Panreac (Barcelona, Spain). 9–Aminoacridine, 2–aminoanthracene, benzo(a)pyrene, D–biotin, D–(+)–glucose monohydrate, glucose–6-phosphate, dimethyl sulfoxide, nicotinamide adenine dinucleotide phosphate, 2–nitrofluorene, and tert–butyl hydroperoxide were purchased from Sigma–Aldrich (St. Louis, MO, USA). L–Histidine monohydrochloride monohydrate was purchased from Merck (Darmstadt, Germany), magnesium sulfate heptahydrate was purchased from (LabChem Inc., Zelienople, PA, USA), nutrient broth nº 2 was from Oxoid (Basingstoke, UK), and sodium azide was purchased from J.T. Baker Chemical Company (Phillipsburg, NJ, USA). Aroclor 1254–induced rat liver S9 was purchased from Trinova Biochem (GmbH, Giessen, Germany). Ultrapure water from a Milli-Q water purification system (Millipore, Molsheim, France) was used to prepare all the solutions, dilutions, and culture media.

### 3.2. Plant Material

Leaves of *Lannea velutina* and *Sorindeia juglandifolia* were collected in October 2015 in Guinea-Bissau. Botanical identification was performed by Dr. Luís Catarino from the Department of Plant Biology, Faculty of Sciences, University of Lisbon. Sample vouchers were deposited in the herbarium LISC of the Universidade de Lisboa (*L. velutina* LCatarino 2131, *S. juglandifolia* LCatarino 2129).

### 3.3. Preparation of Extracts

The dried leaves of both plants were ground and extracted using the maceration method with 70% ethanol at room temperature under agitation. The extracts were concentrated using a rotary evaporator at a temperature of 38 ± 1 °C and freeze-dried. The dried hydroethanolic (70%) extracts (LvLE, SjLE) were stored in a desiccator and protected from light until use.

### 3.4. HPLC-UV/DAD

The extracts were analyzed by HPLC using a Waters Alliance 2690 Separations Module (Waters Corporation, Milford, MA, USA) coupled with a Waters 996 photodiode array detector (UV/DAD) (Waters Corporation, Milford, MA, USA). The Purospher RP-18 end-capped HLPC column (particle size 5 µm, 250 × 4 mm) was connected to a pre-column with the same stationary phase. A mixture of water + 0.1% formic acid (solvent A) and acetonitrile (solvent B) was used as the mobile phase. The injection volume was 25 µL with a flow rate of 1 mL/min. A solvent gradient of 95:5 to 0:100 in 60 min was used.

Before the analysis, samples were solubilized in water (10 mg/mL) and filtered through a polytetrafluoroethylene syringe filter (0.2 µm). Data were collected and analyzed using Waters Millennium^®^ 32 Chromatography Manager (Waters Corporation, Milford, MA, USA). The chromatogram was monitored and registered on Maxplot wavelength (240–650 nm).

### 3.5. Repeated-Dose Toxicity (28 Days)

#### 3.5.1. Animals

Male CD–1 mice (5–10 weeks old, 28–39 g) were purchased from the Institute of Hygiene and Tropical Medicine (IHMT), Universidade NOVA de Lisboa. The animals were housed and acclimatized for seven days at the Animal Facility of the Faculty of Pharmacy, Universidade de Lisboa, under the following conditions: temperature 22 ± 3 °C, relative humidity of 40–60%, and 12 h light/dark cycle. The animals received standard laboratory chow (4RF21 GLP; Mucedola srl, Milan, Italy) as food and had free access to water.

All procedures were conducted in agreement with the animal welfare committee of the Faculty of Pharmacy, Universidade de Lisboa (protocol CEEE-002/16 approved by the Ethics Committee for Animal Experiments (CEEA) in 2016, representing the expert national authority “Direção Geral de Alimentação e Veterinária” (DGAV) according to the EU Directive (2010/63/UE) and Portuguese laws (DR 113/2013, 2880/2015, and 260/2016). Additionally, all experiments were conducted according to ARRIVE Guidelines for Reporting Animal Research.

#### 3.5.2. Experimental Protocol

The 28-day oral repeated dose toxicity study was conducted following OECD guideline no. 407 [25]. Male CD-1 mice (n = 6) were randomly allocated to a control group, which received distilled water, and three dosage groups (D1–D3). The low-dose group (D1) received 50 mg SjLE/kg of body weight (bw); the mid-dose group (D2) received 400 mg SjLE/kg bw; and the high-dose group (D3) received 1000 mg SjLE/kg bw.

The SjLE was orally administered for 28 days using an intragastric tube (10 mL/kg bw) in single doses of 50, 400, and 1000 mg/kg bw. The bw of each animal was recorded once weekly. Before and after each administration, and twice a day during all the experimental procedures, animals were monitored for signs of toxicity (changes in appearance, posture, response to handling, occurrence of autonomic activity) and mortality. Additionally, the general behavior and food consumption of each group of animals were recorded throughout the study period. At the end of the experiment, all animals were anesthetized with a mixture of ketamine: xylazine (100 mg/kg:10 mg/kg), and blood samples were collected via cardiac puncture for biochemical analyses. After blood collection, the animals were euthanized by anesthetic overdose, and their organs were collected for further histopathological macroscopic and microscopic studies (if needed).

#### 3.5.3. Biochemical Analysis

Samples of blood were collected in tubes and centrifuged (4000 rpm, 15 min) to separate the serum. The serum samples were stored at −20 °C until analysis.

Serum biochemical parameters, such as pancreatic biomarkers (amylase and lipase), hepatic biomarkers (alanine aminotransferase [ALT] and aspartate aminotransferase [AST]), renal biomarkers (creatinine and urea), and lipidic profile biomarkers (total cholesterol [TC], high-density lipoprotein [HDL–cholesterol], low-density lipoprotein [LDL–cholesterol], very–low–density lipoprotein [VLDL-cholesterol], and triglycerides) were evaluated at the laboratory of Clinical Analysis Joaquim Chaves certified under the ISO9001–2015 standard. The analytical determinations were made according to the protocols described by ref. [29].

### 3.6. Bacterial Reverse Mutation Assay (Ames Test)

The Ames test was carried out following OECD guideline No. 471 [24] and the ICH S2 (R1) guideline [29], according to previously published protocols [37,46].

Five *Salmonella enterica* serovar Typhimurium strains were used: TA98, TA100, TA102, TA1535, and TA1537. The TA100, TA102, and TA1535 strains were used for the detection of mutagens that cause base-pair substitution mutations, while the TA98 and TA1537 strains were used for the detection of frameshift mutagens [27,37,38].

The study was conducted using the direct plate incorporation method with and without metabolic activation. The strains were inoculated into nutrient broth medium and incubated for 12–16 h, at 37 °C, in the dark, with shaking at 210 rpm in an orbital incubator, and kept at 4 °C until use.

S9 mix (10%, *v*/*v* rat liver S9, 0.4 M MgCl2, 1.65 M KCl, 1 M glucose–6–phosphate, 0.1 M nicotinamide adenine dinucleotide phosphate, and 0.2 M sodium phosphate buffer, pH 7.4) was freshly prepared and kept on ice during the experiment.

The extracts were dissolved in DMSO (up to 40%), which also served as the negative control, and concentrations of 250, 625, 1250, 2500, 3750, and 5000 µg/plate were tested for mutagenicity with and without metabolic activation.

Briefly, different volumes (10, 25, 50, 100, 150, and 200 µL [assay without metabolic activation] and 25, 50, 100, and 200 µL [assay with metabolic activation]) of each extract up to 200 µL (completed with DMSO 40%) were mixed with 500 µL sodium phosphate buffer (0.1 M, pH 7.4) (assay without metabolic activation) or S9 mix (assay with metabolic activation), 100 µL of the bacterial culture, and 2 mL of melted top-agar, supplemented with 0.05 mM biotin and histidine, at 45 °C. This mixture was then vortexed and plated on Petri dishes with Vogel-Bonner agar medium, supplemented with 2% glucose.

After a 48 h incubation at 37 °C, manual counting of His^+^ revertant colonies for each concentration was performed.

All assays were performed in triplicate. The results were expressed as the mean number of revertant colonies with the standard deviation (mean ± SD). The positive controls were sodium azide (SA), 2–nitrofluorene (2-NF), 9–aminoacridine (9–AA), and tert-butyl hydroperoxide (tBHP) for the assay without metabolic activation, and 2–aminoathracene (2-AA) and benzo(a)pyrene (B*a*P) for the assay with metabolic activation (Table 3).

### 3.7. Data Analysis

The experimental results were presented as the mean ± SD. The statistical analysis was conducted using Graph Pad Prism 6.01 scientific software. Assuming data normality, parametric one-way analysis of variance (ANOVA) followed by Dunnet’s multiple comparison test was applied, and a *p*-value less than 0.05 (*p* ≤ 0.05) was considered significant.

## 4. Conclusions

Considering the obtained results, we can conclude that the repeated administration of *S. juglandifolia* hydroethanolic extract for 28 days was safe, as it did not cause death or clinical signs of toxicity in any animal at any of the doses tested (50, 400, and 1000 mg/kg bw). Additionally, neither the hydroethanolic extract of *L. velutina* nor the hydroethanolic extract of *S. juglandigfolia* showed mutagenic potential since they did not cause an increase in the number of revertant colonies at any concentration tested up to the highest concentration (5 mg/plate) recommended by the regulatory guidelines, with and without metabolic activation. Overall, these findings suggest that *S. juglandifolia* hydroethanolic extract poses no health or genotoxic risks during short-term consumption and *L. velutina* hydroethanolic extract also poses no genotoxic threat under the conditions of our study.

Although further studies are needed to complete the safety profile of these two medicinal plants, the obtained negative results provide relevant contributions to their toxicological profiles and important contributions to the use of *L. velutina* and *S. juglandigfolia* as therapeutic resources in the future.

## Figures and Tables

**Table 1:** Number of revertant colonies induced by *Lannea velutina* leaf hydroethanolic extract in *S.* Typhimurium strains, with and without metabolic activation.

*Lannea velutina* leaf hydroethanolic extract (70%): number of revertant colonies without metabolic activation, mean (n = 3), and standard deviation (SD)					
**µg/plate**	**TA 98**	**TA 100**	**TA 102**	**TA 1535**	**TA 1537**
250	34 ± 1	149 ± 13	272 ± 3	14 ± 1	11 ± 3
625	36 ± 3	131 ± 6	269 ± 3	14 ± 1	9 ± 2
1250	40 ± 2	117 ± 8	270 ± 2	14 ± 1	7 ± 2
2500	46 ± 3	125 ± 6	267 ± 2	11 ± 4	5 ± 1
3750	41 ± 2	119 ± 2	262 ± 5	9 ± 5	4 ± 1
5000	37 ± 2	123 ± 4	210 ± 6	5 ± 3	10 ± 2
NC	27 ± 3	147 ± 18	276 ± 10	15 ± 4	9 ± 3
PC	488 ± 30	1048 ± 43.2	881± 26	827 ± 13	1354 ± 45
PCr	a	b	c	a	d
*Lannea velutina* leaf hydroethanolic extract (70%): number of revertant colonies with metabolic activation, mean (n = 3), and standard deviation (SD)					
625	54 ± 1	164 ± 1	149 ± 8	17 ± 1	18 ± 1
1250	55 ± 6	179 ± 10	182 ± 1	17 ± 1	15 ± 3
2500	66 ± 1	175 ± 7	165 ± 8	13 ± 2	13 ± 1
5000	58 ± 7	158 ± 1	167 ± 16	16 ± 1	6 ± 1
NC	47 ± 4	157 ± 6	172 ± 2	11 ± 2	12 ± 1
PC	832 ± 35	947 ± 148	732 ± 12	266 ± 1	306 ± 50
PCr	e	f	e	e	e

Abbreviations: NC (Negative control); PC (Positive Control); PCr (Positive Control reference): (a) 2-NF (2-nitrofluorene); (b) SA (sodium azide; (c) tBHP (tert-butyl hydroperoxide); (d) 9-AA (9-aminoacridine); (e) 2-AA (2-aminoanthracene (f) BaP (benzo(a)pyrene).

**Table 2:** Number of revertant colonies induced by *Sorindeia juglandifolia* leaf hydroethanolic extract in *S.* Typhimurium strains, with and without metabolic activation.

*Sorindeia juglandifolia* leaf hydroethanolic extract (70%): number of revertant colonies without metabolic activation, mean (n = 3), and standard deviation (SD)					
**µg/plate**	**TA 98**	**TA 100**	**TA 102**	**TA 1535**	**TA 1537**
250	25 ± 7	144 ± 6	261 ± 2	15 ± 2	16 ± 3
625	29 ± 4	136 ± 9	265 ± 1	14 ± 2	4 ± 1
1250	39 ± 3	126 ± 5	275 ± 3	12 ± 1	6 ± 1
2500	43 ± 11	120 ± 14	262 ± 2	12 ± 2	8 ± 1
3750	37 ± 4	113 ± 9	284 ± 5	14 ± 1	8 ± 4
5000	31 ± 4	136 ± 12	256 ± 1	13 ± 3	10 ± 1
NC	27 ± 3	147 ± 18	276 ± 10	15 ± 4	9 ± 3
PC	488 ± 30	1048 ± 43	881 ± 26	827 ± 13	1354 ± 5
PCr	a	b	c	a	d
*Sorindeia juglandifolia* leaf hydroethanolic extract (70%): number of revertant colonies with metabolic activation, mean (n = 3), and standard deviation (SD)					
**µg/plate**	**TA 98**	**TA 100**	**TA 102**	**TA 1535**	**TA 1537**
625	54 ± 3	146 ± 18	177 ±1	16 ± 4	15 ± 2
1250	54 ± 8	159 ± 5	179 ± 11	11 ±1	19 ± 1
2500	55 ± 4	156 ± 9	177 ± 13	19 ± 1	19 ±3
5000	46 ± 6	148 ± 14	175 ± 2	16 ± 3	5 ± 1
NC	47 ± 4	157 ± 6	172 ± 2	11 ± 2	12 ± 1
PC	832 ± 35	947 ± 148	732 ± 12	266 ± 1	306 ± 50
PCr	e	f	e	e	e

Abbreviations: NC (Negative control); PC (Positive Control); PCr (Positive Control reference): (a) 2-NF (2-nitrofluorene); (b) SA (sodium azide; (c) tBHP (tert-butyl hydroperoxide); (d) 9-AA (9-aminoacridine); (e) 2-AA (2-aminoanthracene (f) BaP (benzo(a)pyrene).

**Table 3:** Chemicals used as positive controls in the Ames assay with and without metabolic activation.

Strains	Positive Control References without S9	Positive Control References with S9
TA98	2–nitrofluorene (5 µg/plate)	2–aminoanthracene (2 µg/plate)
TA100	sodium azide (1.5 µg/plate)	Benzo(*a*)pyrene (5 µg/plate)
TA102	*Tert*–butyl hydroperoxide (50 µg/plate)	2–aminoanthracene (10 µg/plate)
TA1535	sodium azide (1.5 µg/plate)	2–aminoanthracene (10 µg/plate)
TA1537	9–aminoacridine (100 µg/plate)	2–aminoanthracene (10 µg/plate)

## Data Availability

Not applicable.

## Supplementary Materials

The following supporting information can be downloaded at: https://www.mdpi.com/article/10.3390/plants12010130/s1, Table S1: Effects of the *S. juglandifolia* leaf hydroethanolic extract on body weight and food intake of mice administered daily by gavage with 10 mL/kg of the extract in a single dose for 28 days. Table S2: Behavioral patterns of mice treated with the vehicle control and the different doses of the extract of *S. juglandifolia*.
