# Supplementary material for: Contribution to the Preclinical Safety Assessment of Lannea velutina and Sorindeia juglandifolia Leaves
Source: Plants (Basel). 2022 Dec 27;12(1):130. doi: 10.3390/plants12010130 (PMC9823897; doi:10.3390/plants12010130)
Supplement: Supplementary file 1 [file plants-12-00130-s001.zip › plants-2111125-supplementary.pdf]

Table S1 – Behavioral patterns of mice treated with the vehicle control and the different doses of the extract of *S. juglandifolia*

**Time**		**Parameters**						
		Agitation (no. animals)[1]	Changes in respiration (no. animals)	Lesions (no. animals)[2]	Changes in fur/skin (no. animals)[3]	Changes in eyes (no. animals)	Changes in Mucous membrane (no. animals)	Mortality (no. animals)[4]
8h	CG	N	A	A	A	A	A	A
	D1	N	A	P (1)	P (1)	A	A	A
	D2	P (6)	A	A	A	A	A	A
	D3	N	A	A	A	A	A	A
24h	CG	P (6)	A	P (1)	P (1)	A	A	A
	D1	N	A	P (1)	P (1)	A	A	A
	D2	P (6)	A	A	A	A	A	A
	D3	N	A	A	A	A	A	A
48h	CG	N	A	A	A	A	A	A
	D1	P (6)	A	P (1)	P (1)	A	A	A
	D2	N	A	A	A	A	A	A
	D3	N	A	A	A	A	A	A
7 days	CG	N	A	P	P	A	A	A
	D1	N	A	P	P	A	A	A
	D2	N	A	P	A	A	A	A
	D3	N	A	A	A	A	A	P (1)
15 days	CG	N	A	A	A	A	A	A
	D1	N	A	P (2)	P (1)	A	A	A
	D2	N	A	P (3)	P (3)	A	A	A
	D3	N	A	P (1)	P (1)	A	A	P (1)
28 days	CG	N	A	P (1)	A	A	A	P (1)
	D1	N	A	P (2)	P (2)	A	A	P (1)
	D2	N	A	P (1)	P (1)	A	A	A
	D3	N	A	P (2)	P (2)	A	A	A

Abbreviations: CG- control group; D1 (Dose 1- 50mg/kg); D2 (Dose 2- 400mg/kg); D3 (Dose 3- 1000mg/kg); N-Normal; P- Present; A- Absent; *Number of animals affected in parenthesis.

1. 8 hours after SjLE administration, a small wound with loss of fur was observed in one animal of D1 group.
2. Increase in agitation observed after 24 hours in D2 group; after 24h in Control group and D2 group; after 48h in D1 group.
3. On day 7, two animals in the Control group presented wounds in the back with a loss of fur, one animal in the D1 group had a lesion in the paw with bleeding, and one animal from D2 group presented a small wound on the tail. On day 15, there were 6 animals with wounds/lesions (two in the D1 group, three in the D2 group and 1 in D3 group); Until the last day of the assay more animals with wounds were registered in all groups (one in Control group, two in the D1 group, one in the D2 group and two in D3 group).
4. On day 6 one animal from D3 group was found dead, and a necropsy was made to assess the cause of death, but no sign of toxicity was observed. On day 9, one animal from D3 group died right after administration with SjLE due to misadministration. On days 19 and 20 two animals were found dead (1 in the Control group and another in Dose 1 group) a necropsy was made to assess the cause of death, but no sign of toxicity was observed.

Table S2 – Effects of the *S. juglandifolia* leaf hydroethanolic extract on body weight and food intake of mice administered daily by gavage with 10 mL/kg of the extract in a single dose for 28 days

**Parameters**	**Groups**			
	**Control**	**Dose 1**	**Dose 2**	**Dose 3**
**Initial weight (g)**	31 ± 4.7	36.6 ± 3.2	33.3 ± 2.1	28.5 ± 1.3
**Final weight (g)**	31.8 ± 4.9	36.8 ± 2.3	34 ± 1.7	30 ± 1.4
**Cumulative body weight variation in 28 days (%)**	1.4±1.9	-2.3 ± 2.4	-1.4 ± 3.7	1.5 ± 2.2
**Food intake (g/week)**	66.3 ± 9.3	65.6 ± 9.3	57.6 ± 6.3	52.7 ±5.9

Values are presented as mean ± SD; N=6; No significant differences among all groups ($p<0.005$)
